# Pleckstrin homology-like domain family A, member 3 (PHLDA3) deficiency improves islets engraftment through the suppression of hypoxic damage

**DOI:** 10.1371/journal.pone.0187927

**Published:** 2017-11-09

**Authors:** Naoaki Sakata, Yohko Yamaguchi, Yu Chen, Masayuki Shimoda, Gumpei Yoshimatsu, Michiaki Unno, Shoichiro Sumi, Rieko Ohki

**Affiliations:** 1 Department of Surgery, Tohoku University, Aoba-ku, Sendai, Miyagi, Japan; 2 Divisions of Rare Cancer Research, National Cancer Center Research Institute, Tsukiji, Chuo-ku, Tokyo, Japan; 3 Department of Pancreatic Islet Cell Transplantation, Research Institute, National Center for Global Health and Medicine, Toyama, Shinjuku-ku, Tokyo, Japan; 4 Department of Organ and Tissue Reconstruction, Institute for Frontier Life and Medical Sciences, Kyoto University, Shogoin, Sakyo-ku, Kyoto, Japan; Children's Hospital Boston, UNITED STATES

## Abstract

Islet transplantation is a useful cell replacement therapy that can restore the glycometabolic function of severe diabetic patients. It is known that many transplanted islets failed to engraft, and thus, new approaches for overcoming graft loss that may improve the outcome of future clinical islet transplantations are necessary. Pleckstrin homology-like domain family A, member 3 (PHLDA3) is a known suppressor of neuroendocrine tumorigenicity, yet deficiency of this gene increases islet proliferation, prevents islet apoptosis, and improves their insulin-releasing function without causing tumors. In this study, we examined the potential use of PHLDA3-deficient islets in transplantation. We observed that: 1) transplanting PHLDA3-deficient islets into diabetic mice significantly improved their glycometabolic condition, 2) the improved engraftment of PHLDA3-deficient islets resulted from increased cell survival during early transplantation, and 3) Akt activity was elevated in PHLDA3-deficient islets, especially under hypoxic conditions. Thus, we determined that PHLDA3-deficient islets are more resistant against stresses induced by islet isolation and transplantation. We conclude that use of islets with suppressed PHLDA3 expression could be a novel and promising treatment for improving engraftment and consequent glycemic control in islet transplantation.

## Introduction

Islet transplantation is a useful cell replacement therapy that can restore the glycometabolic function of severe diabetes mellitus (DM) patients. It enables the patient to receive appropriate insulin secretion in response to changes in blood glucose levels, and prevents severe hypoglycemic episodes and diabetic secondary complications including cardiovascular, renal, retinopathic and nervous disorders [1–5]. Furthermore, islet transplantation also enhances kidney graft survival in patients who have received kidney cotransplantation [6]. While the outcome of the islet transplantation has gradually improved [7], this procedure still faces many obstacles. In particular, the supplies of donor pancreata are very limited in Japan, while many recipients need multiple donors for insulin independence [7].

It is known that many transplanted islets failed to engraft. First, transplanted islets suffer from various stresses including immune rejection [8], islet ischemia [9], and thrombotic and inflammatory reactions [10] within a couple of days (i.e. early graft loss). Even if islet engraftment is successful, the islets still have to overcome chronic stresses such as inflammation [11], cholestasis [12] and glucotoxicity due to poor control of blood glucose [13] (i.e. late graft loss). Thus, researchers continue to seek new approaches for overcoming early and late graft loss that may improve the outcome of future clinical islet transplantations.

We have previously found that Pleckstrin homology-like domain family A, member 3 (PHLDA3) is a novel p53—regulated repressor of Akt, and is a novel tumor suppressor of pancreatic neuroendocrine tumors (pNET) [14]. Our recent study showed that knockdown of PHLDA3 induced cellular proliferation and prevented apoptosis of a β cell line challenged with streptozotocin (STZ) [15]. We further observed that pancreatic islets were larger in PHLDA3-deficient mice compared to wild-type [15]. Furthermore, although PHLDA3 acts as a tumor suppressor gene, knockout of PHLDA3 by itself is not oncogenic and does not generate pNETs [15]. We speculated that these characteristics of PHLDA3-deficient islets might make them more suitable for islet transplantation. In this study, we further examined the potential utility of PHLDA3 deficiency in islet transplantation using PHLDA3-deficient mice.

## Materials and methods

### Animals

PHLDA3-deficient and wild-type C57B6 mice were provided by Dr. Ohki, and 8–12 week-old mice were used as islet donors. The generation of PHLDA3-deficient mice was previously reported [16]. C57B6 mice, 12–16 week-old and weighing 25–30 g (CLEA Japan, Inc., Tokyo, Japan), were used as diabetic recipients. The animals were housed under pathogen-free conditions with a 12-hour light cycle and with free access to food and water.

All animal care and treatment procedures were carried out in accordance with our institutional regulations, and the Institutional Animal Care and Use Committee of Tohoku University Graduate School of Medicine and Tohoku University Center for Gene Research approved the experimental protocol (2016MdA-073 and 2016MdLMO-016).

### Genotyping

Sample DNA was extracted from a tip of the tail of PHLDA3-deficient and wild-type mice using sodium hydroxide. The genotyping for PHLDA3 was done by polymerase chain reaction (PCR). The primers were 5’ PHLDA3 primer sequence: 5' GAGAGAGTAGCATCGCACGAGCCTC, center reverse primer sequence: 5' CATTCTCTTCGGATCTACGCTCTGTTG), and neo forward primer sequence: 5' GAGGCTATTCGGCTATGACTGGGCAC.

### Islet isolation

Islets of both PHLDA3-deficient and wild-type mice were isolated by collagenase digestion and purification following a modification of Gotoh’s method [17]. In brief, digestion of pancreas using 1mg / mL collagenase V (Sigma-Aldrich, St. Louis, MO, USA) solution was done at 37°C for 15–20 mins, after which islets were isolated by Ficoll^®^ PM 400 (Sigma-Aldrich) density gradient (1.100, 1.092, 1.086, 1.044) centrifugation at 2,000 rpm for 13 mins. Approximately 100% purity was achieved using a 100 μm Cell Strainer (Corning Inc., Corning, NY, USA) and handpicking under the microscope.

### Assessment of viability and insulin-releasing function of islets

Viability and insulin-releasing function were assessed in PHLDA3-deficient and wild-type islets isolated at three separate times, with five to six mice per isolation.

Islet cell viability and insulin—releasing function at 24 hs after isolation were analyzed to evaluate the preoperative condition of the islets.

Islets were incubated in SYTO Green 11 (Life Technologies Japan, Tokyo, Japan) and ethidium bromide (Sigma-Aldrich) solution (1:500 diluted by phosphate buffer saline (PBS), respectively) at room temperature for 30 mins. Twenty islets per isolation were randomly selected, and fluoroscopic images were captured by confocal microscopy (C2+; Nikon Co., Tokyo, Japan). Viable cells stained green (SYTO Green 11) and dead cells stained red (ethidium bromide). Islet viability was quantified as the percentage of viable cells per total cells (sum of dead and viable cells) per islet. The average of islet cell viability was compared between PHLDA3-deficient and wild-type islets.

Insulin-releasing function in response to glucose stimulation was examined by static incubation of 24 hs-cultured islets in low- and high-concentration glucose solutions. In detail, ten islets from each group were plated onto 8.0 μm pore sized cell culture inserts (product number 353097, Falcon / Corning Inc.) in 24-well plates and incubated in 1 ml of RPMI1640 medium containing low glucose (3.3 mM) for 1 h as a preincubation step. Next, islets were incubated in media containing 3.3 mM glucose for 1 h (termed sample “L”) and then media containing 16.7 mM glucose for 1 h (termed sample “H”). Samples were collected to measure the volume of insulin secretion from cultured islets. Insulin secreted into the culture media was measured using an insulin ELISA kit (Shibayagi Co., Shibukawa, Gumma, Japan). The ratio of insulin secreted into samples H and L was calculated and taken as the stimulation index (SI) [10]. Secreted insulin volume at low and high glucose stimulation and SI were compared between PHLDA3-deficient and wild-type islets.

### Induction of diabetes in recipient mice

Diabetes was induced in recipient mice by intraperitoneal injection of STZ (200 mg/kg body weight; Sigma-Aldrich). The blood glucose (BG) levels of the mice were measured using the Ascensia Breeze II BG monitoring system (Bayer Health Care, Kita, Osaka, Japan) 7 days after the injection. Mice with BG levels exceeding 400 mg/dL were used as diabetic recipients.

### Islet transplantation and grouping

Islet transplantation was done into the liver via the portal vein. In brief, diabetic mice were anesthetized using isoflurane (2% for induction and 0.5–1.5% for maintenance; Forane^®^, Sumitomo Dainippon Pharma Co., Ltd., Osaka, Japan) and then subjected to laparotomy. One hundred fifty islet equivalents (IEQs) were infused into the portal vein (superior mesenteric vein at pancreas) directly using 27G Surshield^™^ Safety Winged Infusion Sets with 300 μL RPMI1640 media. After certifying hemostasis by pressing the portal vein using a cotton swab, the abdominal wall was closed by 5–0 Polysorb suture (Covidien / Medtronic, Minneapolis, MN, USA).

One hundred fifty islet equivalents (IEQs) were transplanted into the liver via the portal vein. The mice, which received the PHLDA3-deficient islets were classified as the KO group (n = 17), and those receiving wild-type islets as the WT group (n = 14).

### Measurements of blood glucose and C-peptide, and glucose tolerance test after transplantation

BG and C-peptide were measured for two months after transplantation. Non-fasting BG was measured at 8–10 a.m. on postoperative days (PODs) 0 (i.e. pretransplantation), 1, 3, 5, 7, 10, 14, 17, 21, 24, 28, 35, 42, 49 and 56. Normoglycemia was defined as a BG level < 200 mg/dL. Blood samples for C-peptide were collected from tail vein of the mice on PODs 0, 3, 7, 14, 28 and 56. The collected blood volume was 150 μL (approximately 70 μL of serum was obtained from the blood sample). The samples were isolated into serum and preserved at -80°C until measurement. C-peptide levels were measured using a mouse C-peptide ELISA kit (Shibayagi Co.). Intraperitoneal glucose tolerance tests (IPGTTs) were also performed on PODs 29 and 57. Glucose solution (2 g/kg body weight) was injected into the peritoneal cavity of the mouse after 10–12 hrs fasting, and glucose levels measured at 0, 30, 60, 90 and 120 min after the injection. Area under the curves (AUCs) in GTTs (AUC-GTT) were calculated to compare between KO and WT groups.

### Histological assessment

The livers of mice in the KO and WT groups were recovered at 12 hs after transplantation to evaluate any early changes in transplanted islets (5 KO mice and 6 WT mice) and at 56 days after transplantation to evaluate engraftment of islets (16 KO mice and 14 WT mice). In addition, one mouse from the KO group was recovered at 180 days after transplantation. The livers were fixed using 10% formalin neural buffer solution and embedded using paraffin. Paraffin sections (5 μm) of specimens were either stained with hematoxylin and eosin (HE, to detect necrosis of islets) or examined by immunohistochemistry for insulin (to detect islets), Ki67 (to detect proliferation of the endocrine cells in islets) and HIF-1α (to prove cellular proliferation and survival via hypoxia). The primary antibodies were mouse anti-insulin (1:1000; K36AC10, Sigma-Aldrich), rabbit anti-Ki67 antibody (clone SP6; Nichirei Biosciences Inc., Tokyo, Japan) diluted 1:200 and rabbit anti- hypoxia-inducible factor -1α (HIF-1α) antibody (1:50; H-206, Santa Cruz Biotechnology Inc., Dallas, TX, USA). After incubation with the primary antibodies, the specimens were incubated with the biotinylated secondary immunoglobulin—G antibody (Nichirei Biosciences). A peroxidase substrate solution containing 3,3’ -diaminobenzidine (Dako Japan, Tokyo, Japan) was used for visualization. Apoptosis was quantified using the terminal deoxynucleotidyl transferase-mediated deoxyuridine triphosphate-biotin nick end labeling (TUNEL) assay, the Peroxidase *In Situ* Apoptosis Detection Kit (EMD Millipore, Billerica, MA, USA), and a Discovery XT automated staining system (Ventana Medical Systems, Inc., Tucson, AZ, USA). All of the histological figures were observed using a BZ-9000 microscope (KEYENCE Co., Tokyo, Japan), and these findings were quantified for statistical evaluation. The areas of engrafted islets at POD 56 were calculated using insulin-stained specimen. Necrosis in islets was assessed by quantifying the percentage of necrotic area (strong eosin-stained area without viable cells) in an islet. Apoptosis and cellular proliferation were assessed by the percentage of TUNEL- and Ki67-positive cell numbers per total cells in an islet. The assessment of HIF-1α was done by density of HIF-1α-positive islet cells, and the ratio of the density between islet and HIF-1α negative area in liver tissue. These quantifications were done using ImageJ^®^ software (National Institutes of Health, Bethesda, MD, USA).

### Hypoxia treatment

To evaluate the influence of hypoxic conditions on the transplanted islets, we subjected islets from PHLDA3 WT and KO mice to hypoxic culture conditions using a BIONIX-1 hypoxic culture kit (Sugiyama-Gen Co., Ltd., Tokyo, Japan) [18], which consisted of an deoxidizing agent (Sugiyama-Gen), an OXY-1 oxygen monitor (JIKCO, Tokyo, Japan), a plastic bag (Mitsubishi Gas Chemical, Tokyo, Japan), and plastic clips for sealing the bag. In detail, isolated KO or WT islets were divided into normoxic or hypoxic cultures. KO and WT islets in normoxic culture were incubated in at 37°C in 5% CO_2_ / 20% O_2_ for 6 hs (named KO or WT normal group). KO and WT islets in the hypoxic culture were packed into the plastic bag with a deoxidizing agent and oxygen monitor. When the content of O_2_ was decreased to 1%, islets were isolated from the deoxidizing agent using the sealing clip. The islets were incubated at 37°C in 5% CO_2_ for 6 hs, as same as KO and WT normal groups (named KO or WT hypoxia group). Four hundred IEQs were used for one group. After normoxic and hypoxic culture, the islets were rinsed using phosphate buffer saline (PBS), and used for Western blotting.

### Western blotting analysis

Islet cells were lysed in sodium dodecyl sulfate (SDS)-sample buffer [0.1 M Tris-HCl pH 6.8, 2% SDS, 10% (v/v) Glycerol, 0.01% Bromophenol blue] and subjected to Western blotting. The antibodies used for this assessment are described in S1 Table.

### Statistics

Insulin-releasing function, BG, blood C-peptide, and BG in the IPGTT were compared between the PHLDA3-knockout and wild-type groups using two-way repeated measures analysis of variance (ANOVA). Viability of islets, AUC of BG change in the IPGTT, numbers and area of the engrafted islet, the percentages of apoptosis, necrosis and cellular proliferation, density of HIF-1α positive islet cells, and ratio of density of HIF-1α were compared between them using non-parametric analysis Mann–Whitney U test. All the data are presented as the mean ± standard error of the mean (SEM). α level 0.05 was provided to all the statistical assessments. All the tests were two-sided. All the statistical analyses were done by JMP^®^12.0.0 (SAS Institute Inc., Cary, NC, USA).

## Results

### PHLDA3-deficient islets are resistant against stress caused by the islet isolation process

**Fig 1:**
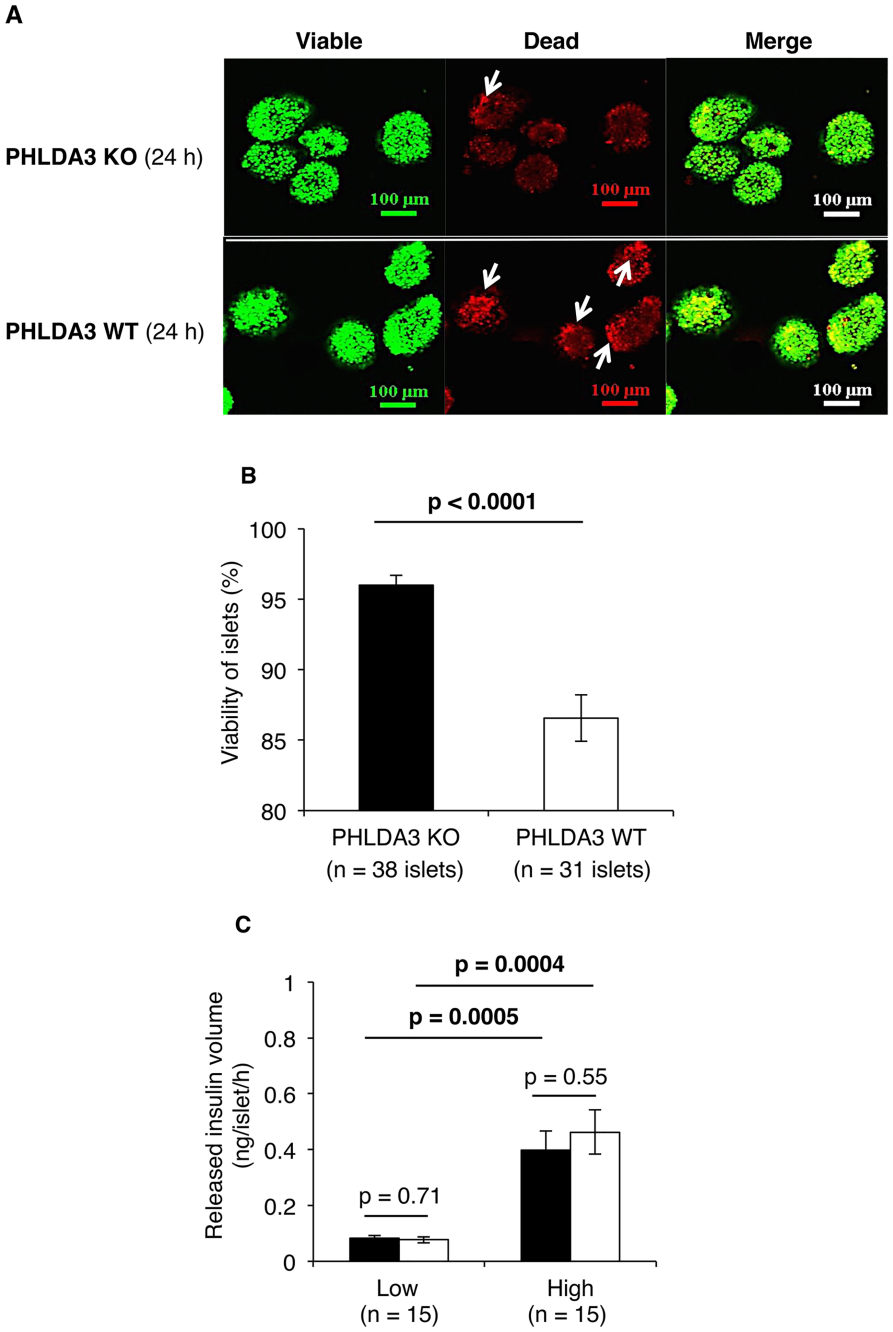
Condition of PHLDA3-deficient islets at 24 hs after isolation. **A.** Islets from PHLDA3 KO and WT groups were stained by SYTO Green 11 (for viable cells) and ethidium bromide (for dead cells, indicated by arrow). More viable cells and fewer dead cells were seen in KO group than the WT group. The size of the scale bar is 100 μm. **B.** Quantification of islet viability showed significantly higher viability for PHLDA3-deficient (= KO: n = 38 islets) than wild-type islets (n = 31 islets). **C.** Volumes of released insulin following stimulation in low (3.3mM) and high (16.5mM) glucose solutions. Insulin release dependent on glucose concentration was seen in both the KO and WT groups, and the levels between the two groups were similar (n = 15 islets in both groups). For statistical analysis, Mann–Whitney U test was used for viability and two-way repeated measures ANOVA for insulin—releasing function.

Fig 1 shows PHLDA3-deficient (n = 38 islets) and wild-type islets (n = 31 islets) cultured for 24 hs and incubated with fluorescent dyes to stain viable and dead cells. The regions containing dead cells in the PHLDA3-deficient islets were smaller than those of the wild-type islets (Fig 1A), and the calculated viability of PHLDA3-deficient islets was also higher than wild-type islets (96.0 ± 0.7% vs. 86.6 ± 1.6%: p < 0.0001; Fig 1B).

We further observed no differences in the insulin-releasing functions between the PHLDA3-deficient and wild-type islets (n = 15 islets in both groups). Insulin release as a function of glucose concentration was similar in both groups (PHLDA3-deficient islets: 0.08 ± 0.01 ng/islet/h at low glucose and 0.40 ± 0.07 ng/islet/h at high glucose, p = 0.0005; wild-type islets: 0.08 ± 0.01 ng/islet/h at low glucose and 0.46 ± 0.08 ng/islet/h at high glucose, p = 0.0004, Fig 1C).

### Metabolic condition was significantly improved by PHLDA3-deficient islet transplantation

**Fig 2:**
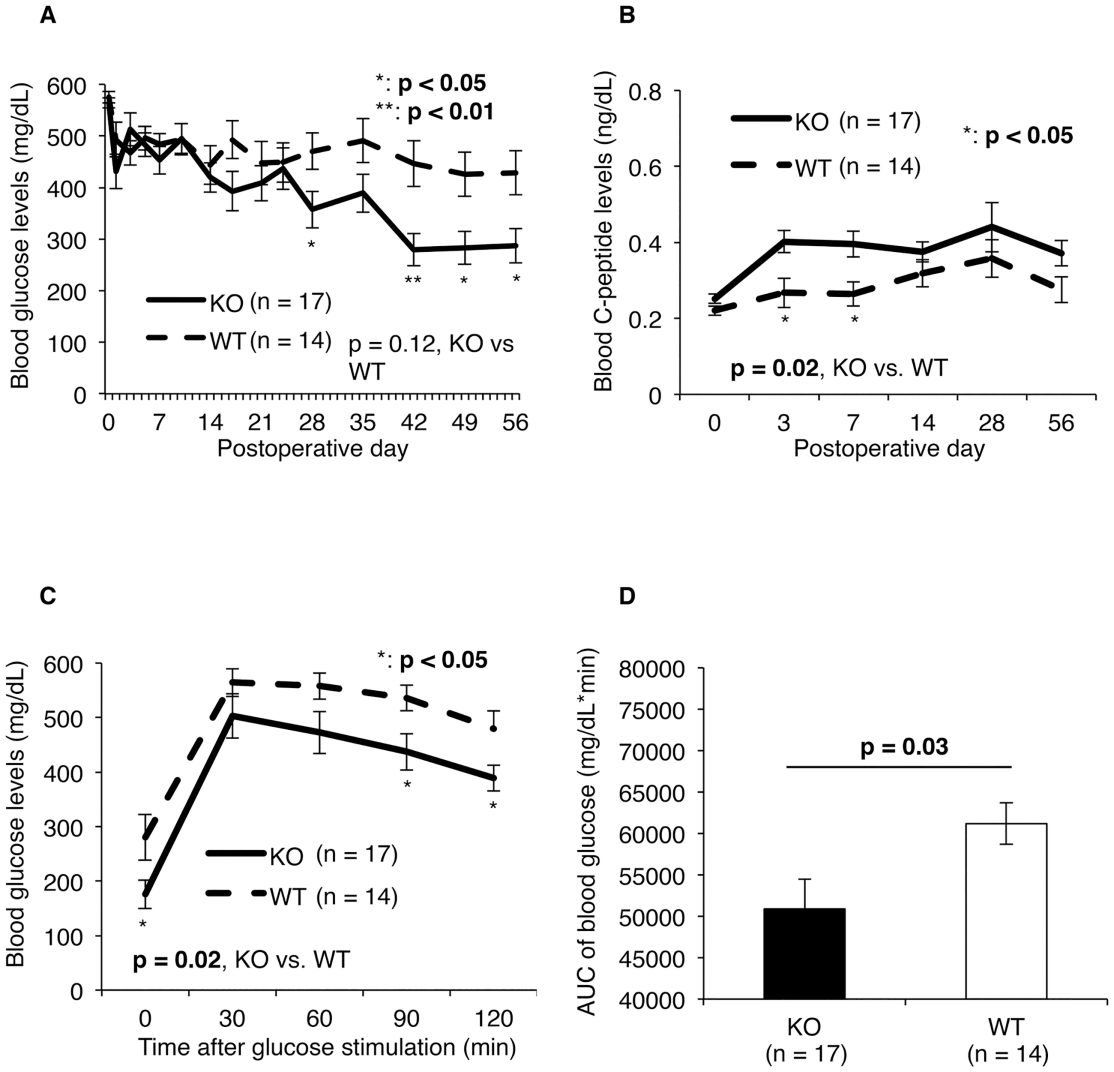
Metabolic condition of diabetic mice after intrahepatic islet transplantation with PHLDA3-deficient or wild-type islets. Blood glucose (**A**) and C-peptide (**B**) levels were measured after transplantation of PHLDA3-deficient or wild-type islets, and the difference between the KO (n = 17) and WT (n = 14) groups was statistically assessed by two-way repeated measures ANOVA. Levels of both were significantly improved in KO group in comparison with WT group. Blood glucose levels gradually decreased in both groups, but were significantly lower in the KO group compared to the WT group by postoperative day (POD) 28 (357.3 ± 35.7 mg/dL in KO group vs. 470.4 ± 35.4 mg/dL in WT group at POD 28, p = 0.04; 286.8 ± 33.3 mg/dL vs. 428.6 ± 42.4 mg/dL at POD 56, p = 0.02; **A**). Change in blood glucose levels after glucose stimulation (**C**) and AUC of the blood glucose levels after glucose stimulation (**D**) at POD 56 also revealed that the metabolic condition of the mice transplanted with PHLDA3-deficient islets was improved. The times of experiments were eight. For statistical analyses, the two-way repeated measures ANOVA for change of blood glucose level and Mann–Whitney U test for AUC were used.

Fig 2 shows the metabolic conditions of recipient mice transplanted with PHLDA3-deficient (KO; n = 17) or wild-type (WT; n = 14) islets. Blood glucose levels gradually decreased in both groups, but were significantly lower in the KO group (357.3 ± 35.7 mg/dL) compared to the WT group (470.4 ± 35.4 mg/dL) by postoperative day (POD) 28 (Fig 2A). The blood C-peptide levels in the KO group was also higher than in the WT group during the observation period (p = 0.02; Fig 2B). In particular, an obvious increase in C-peptide levels was seen in the KO group at PODs 3 and 7 (0.40 ± 0.03 ng/mL vs. 0.27 ± 0.04 ng/mL at POD 3, p = 0.01; 0.40 ± 0.03 ng/mL vs. 0.26 ± 0.03 ng/mL at POD 7, p = 0.01; Fig 2B). Moreover, blood glucose levels after glucose stimulation at POD 56 were lower in the KO group (p = 0.02, Fig 2C), with especially prominent decreases seen at 90 and 120 mins after glucose stimulation (437.4 ± 33.5 mg/dL vs. 536.3 ± 23.5 mg/dL at 90 min, p = 0.04; 389.3 ± 23.9 mg/dL vs. 479.8 ± 32.9 mg/dL at 120 min, p = 0.04, Fig 2C). The area under the curve of blood glucose was also lower in the KO group (50876.3 ± 3554.8 mg/dL*min vs. 61170.0 ± 2515.0 mg/dL*min, p = 0.03, Fig 2D).

### Conditions of PHLDA3-deficient islets in the early stages of transplantation

**Fig 3:**
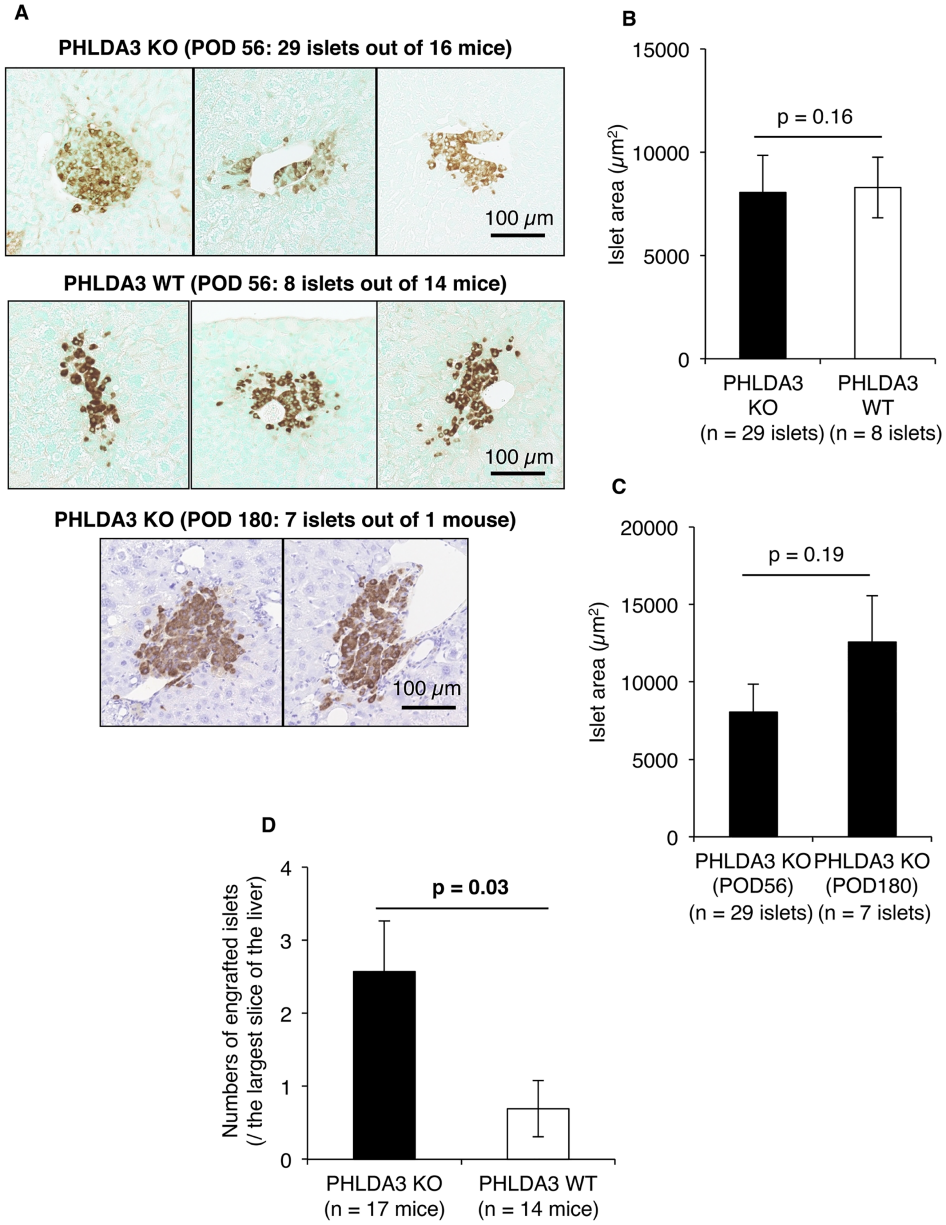
Size and number of engrafted PHLDA3-deficient islets. **A.** Engrafted islets at POD 56 and 180. The size was almost the same between the KO and WT groups at POD 56 (upper and middle lanes). Hyperplasia of PHLDA3-deficient islets was not detected at POD 180, but the size tended to be wider than that at POD 56 (bottom vs top panels). The size of the scale bar was 100 μm. **B** and **C**. Quantification of islet areas. There was no difference between the KO (n = 29 islets) and WT (n = 8 islets) groups at POD 56 (**B**). There was also no difference in sizes between POD 56 (n = 29 islets) and 180 (n = 7 islets) in the KO group, but the islet area at 180 tended to be wider than that at POD 56 (**C**). **D**. Numbers of engrafted islets at POD 56. More engrafted islets were seen in the KO group (n = 17) than the WT group (n = 14). Mann–Whitney U test was used for statistical assessment.

We observed that none of the PHLDA3-deficient islets developed tumors after transplantation (n = 36 islets out of 17 mice, Fig 3A). Our previous study revealed that the size of PHLDA3-deficient islets were larger than wild-type islets [15]. However, in this study, we observed no hyperplasic islets in the KO group (Fig 3A) and no difference between KO (n = 29 islets out of 16 mice) and WT (n = 8 islets out of 14 mice) in the areas of the engrafted islets at POD 56 (8047.3 ± 1801.5 μm^2^ vs. 8289.3 ± 1468.5 μm^2^; p = 0.16; Fig 3B). On the other hand, comparing islet size between POD 56 and POD 180, we observed that islet size in the KO group (n = 7 islets out of 1 mouse) tended to be larger at POD 180 compared to POD 56 (12566.6 ± 3006.0 μm^2^ vs. 8047.3 ± 1801.5 μm^2^; p = 0.19; Fig 3C). We further confirmed greater numbers of PHLDA3-deficient islets succeeded in engraftment compared to wild-type islets at POD 56 (2.6 ± 0.7 islets / the largest slice area of the liver vs. 0.7 ± 0.4 islets / the largest slice area of the liver, p = 0.03, Fig 3D).

**Fig 4:**
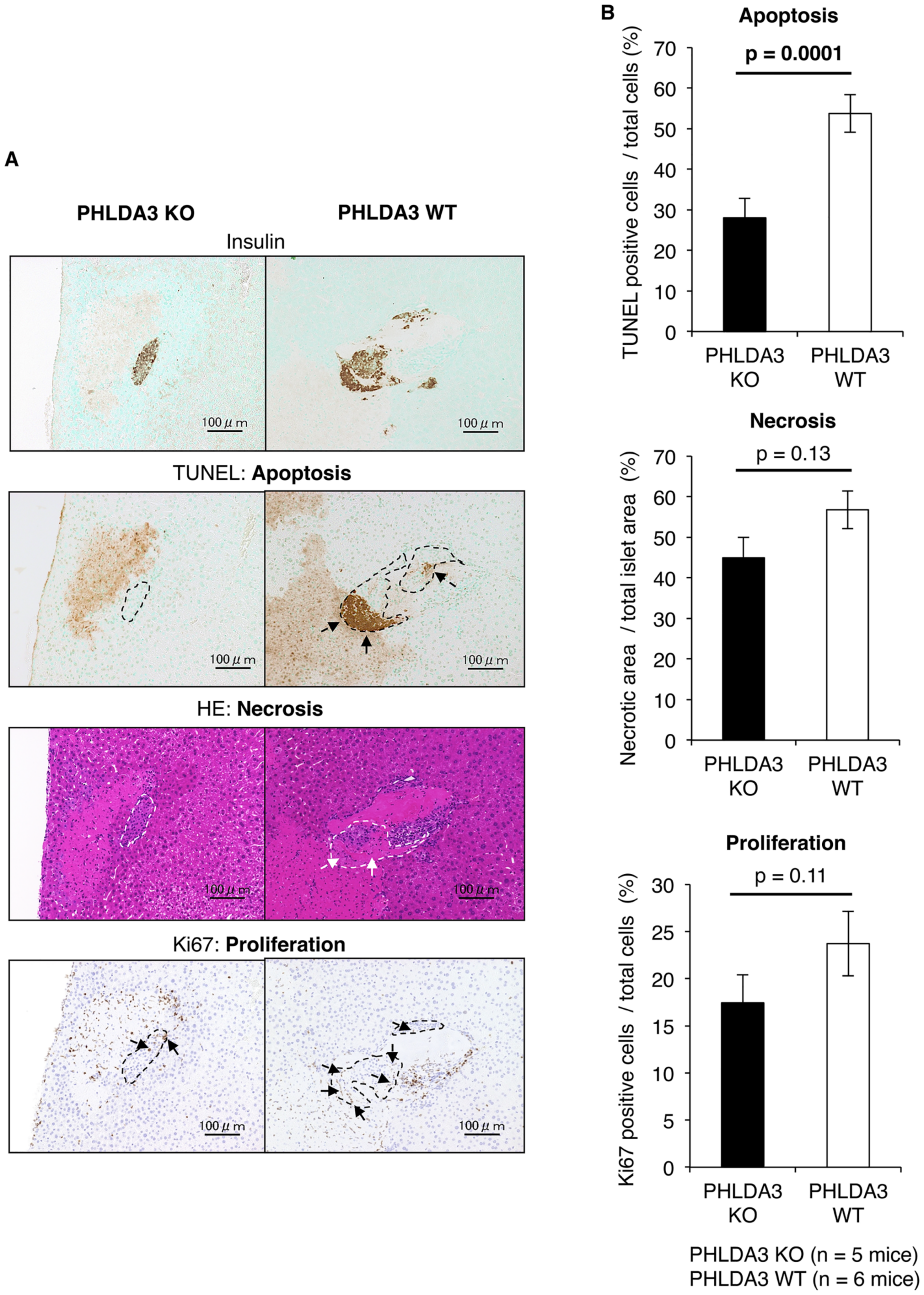
Condition of islets at 12 hs after transplantation. **A**. Histological analyses of KO and WT islets at 12 hs after transplantation. Apoptosis (TUNEL-positive), necrosis (eosin-positive without nucleus area) and proliferation (Ki67-positive) were seen in both the KO (n = 5) and WT (n = 6) groups, but they were less prominent in the KO group. The images (insulin, TUNEL, HE, Ki67) were of the same location. The dashed lines indicate the border of the transplanted islets. The size of the scale bar was 100 μm. **B**. Quantification of the digitalized images showing apoptosis, necrosis and proliferation. The data confirms that apoptosis of transplanted islets at 12 hs was lower in the KO group. The statistical analysis was done using the Mann–Whitney U test.

Next, to further clarify why many of the PHLDA3-deficient islets were successfully engrafted, we histologically evaluated PHLDA3-deficient islets at 12 hs after transplantation. TUNEL-positive islet cells (i.e. apoptotic cells) and eosin-positive areas without nucleus in HE-stained islets (i.e. necrotic areas) were seen in both the KO and WT groups, but these features appeared less numerous in the KO group (Fig 4A). Quantification of these samples revealed that apoptosis and necrosis of the PHLDA3-deficient islets were indeed lower, especially islet apoptosis (28.03 ± 4.84% vs. 53.73 ± 4.61% TUNEL-positive cells: p = 0.0001; 44.93 ± 5.09% vs. 56.77 ± 4.65% necrotic areas in islets; p = 0.13; Fig 4B). Interestingly, cell proliferation (Ki67-positive cells) tended to be seen more in islets in the WT than KO group (17.44 ± 3.00% vs. 23.71 ± 3.41% Ki67-positive cells: p = 0.11; Fig 4A and 4B).

### Activation of the PI3K / Akt pathway in PHLDA3-deficient islets under hypoxic conditions

**Fig 5:**
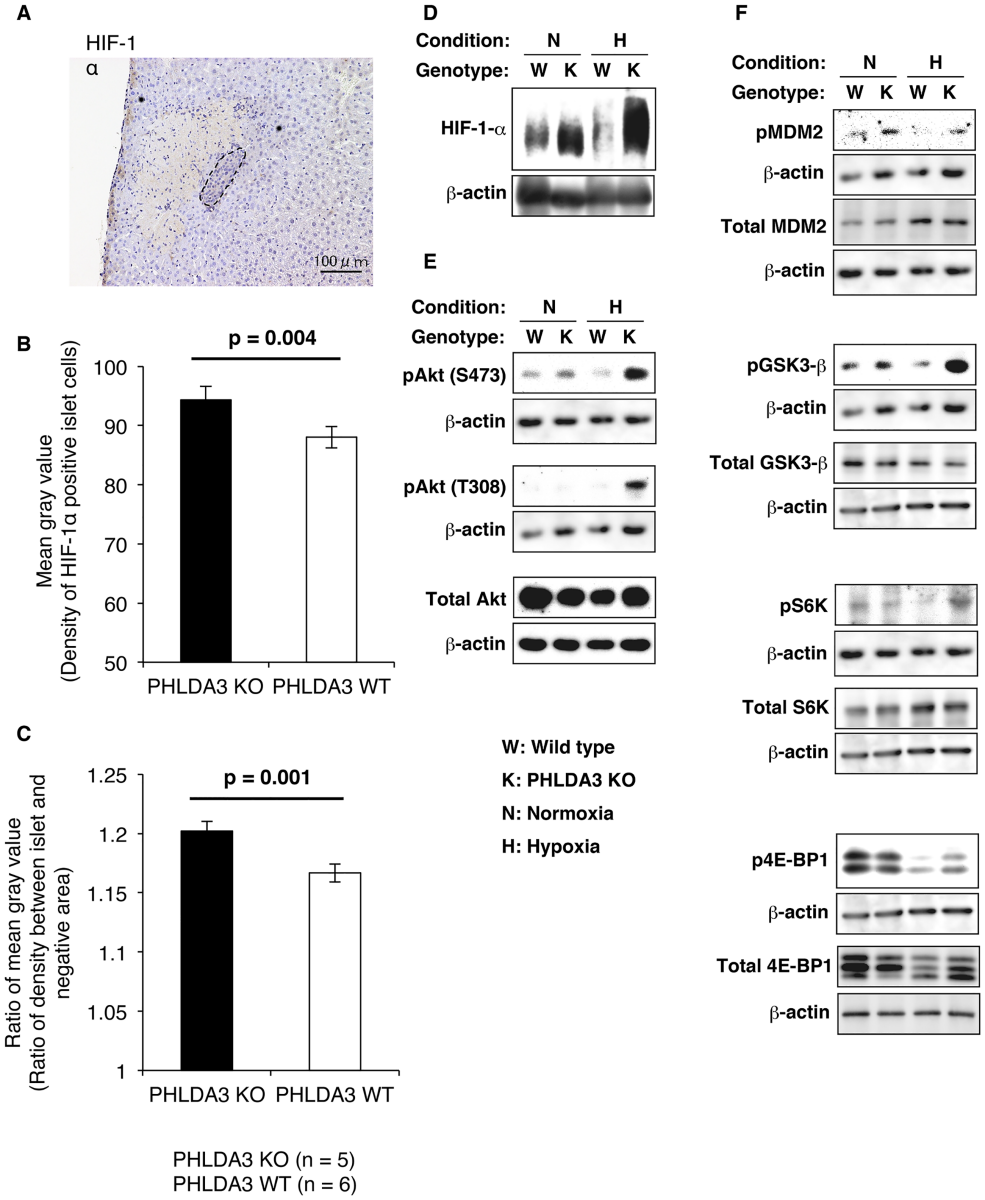
Expression of HIF-1α in transplanted islets at 12 hs after transplantation and Western blotting of islets cultured under hypoxic conditions. **A**. Histological analysis of transplanted PHLDA3-deficient (KO) islets. The expression of HIF-1α was not strong, but relatively higher than in the surrounding liver tissue. The dashed line indicates the border of the transplanted islet. The size of the scale bar was 100 μm. B and C. Density of HIF-1α—positive islet cells (**B**) and ratio of density between islet and HIF-1α-negative area (**C**). The numbers of mice were 5 in the KO group and 6 in the WT group. These data revealed that the expression of HIF-1α was significantly stronger in KO group. The statistical analysis was done using the Mann–Whitney U test. **D-F**. Expression of HIF-1α (D), total and phosphorylation (p) of Akt (E), total and phosphorylation (p) of Akt substrates (F, MDM2, GSK3-β, S6K and 4E-BP1) in PHLDA3-deficient (K) and wild-type (W) islets in normoxic (N) and hypoxic (H) conditions were assessed by Western blotting. Expression of β-actin is also assessed. Same β-actin blot appears for pAkt(S473) and pS6K, pAkt(T308), pMDM2 and pGSK3-β, and total GSK3-β, total S6K and p4E-BP1, since they derive from the same blot. Akt was strongly phosphorylated in PHLDA3-deficient islets under hypoxic conditions. Phosphorylation of Akt-substrate MDM2 and GSK3-β were also induced in PHLDA3-deficient islets. HIF-1α was stabilized in PHLDA3-deficient islets, especially under hypoxic conditions.

Fig 5A to 5C shows the expression of HIF-1α in transplanted PHLDA3-deficient and wild-type islets. Expression was not very high in either the KO (Fig 5A) or WT groups, however the staining density of the HIF-1α—positive islet cells between KO and WT groups was somewhat higher in the KO group (Density of HIF-1α—positive islet cells was 94.3 ± 2.3 in KO group and 88.0 ± 1.8 in WT group, p = 0.004, Fig 5B; Ratio of density between islet and HIF-1α negative area was 1.202 ± 0.008 in KO group and 1.167 ± 0.008 in WT group, p = 0.001, Fig 5C). These data revealed that the expression of HIF-1α was comparatively higher in the transplanted PHLDA3-deficient islets.

Fig 5D–5F shows the activity of the Akt pathway assessed by Western blotting of lysates from PHLDA3-deficient (KO) and wild-type (WT) islets cultured under normoxic and hypoxic conditions. Hypoxia induced higher levels of HIF-1α expression in the PHLDA3-KO islets (Fig 5D). Akt activity (phosphorylation of Akt at S473 and T308) was also elevated in PHLDA3-KO islets under both normoxic and hypoxic conditions. Notably, Akt activity was especially strong under hypoxic conditions (Fig 5E). Moreover, phosphorylation of representative Akt substrates, glycogen synthase kinase 3 -β (GSK3-β, associated with glucose storage and uptake) and mouse double minute protein 2 (MDM2, which negatively regulates p53 the tumor-suppressor activity), were increased in PHLDA3-KO islets compared to WT islets under both normoxic and hypoxic conditions. In addition, phosphorylation of 2 targets of mTOR, p70S6K and 4eBP1, were increased in PHLDA3-KO islets compared to WT islets under hypoxic conditions (Fig 5F).

## Discussion

The clinical outcome of islet transplantation currently suffers from an unacceptable level of graft rejection and there is a great need to find novel and reliable donor sources to improve this treatment in the future. Many candidate donor sources have been explored, including stem cell-derived islet cells [19–21] and xenogeneic islets of large animals [22], however some modifications will be necessary to utilize these cellular sources. In particular, prevention of the early graft loss is a major key factor in the success of islet transplantation.

Our work has focused on the unique characteristics of PHLDA3, a p53-regulated repressor of Akt and a novel suppressor of pNETs. PHLDA3 competes with the Pleckstrin homology (PH) domain of Akt for binding to membrane lipids and inhibits Akt translocation to the cellular membrane, thereby preventing its activation [14]. Our previous study revealed that PHLDA3 is a tumor suppressor, inactivation of which causes the development of pNET through the upregulation of the PIP3 / Akt signaling pathway [15]. First, high frequency of loss of heterozygosity (LOH) at PHLDA3 gene was seen in pNET patients, and this LOH is correlated with poor prognosis. In addition, the PHLDA3 promoter is methylated at a high frequency in pNET patients [14]. These observations suggested a two-hit mechanism (LOH and methylation) by which the PHLDA3 gene is inactivated, leading to the development of pNET.

PHLDA3 represses Akt activity and Akt-regulated biological processes in pancreatic islets. Activation of the PI3K / Akt pathway induces various biological processes including insulin-mediated glucose transport, protein and glycogen synthesis, proliferation, cell growth, differentiation, and survival [23]. Therefore, deficiency of PHLDA3 results in islet hyperplasia and improvement in insulin-releasing function. PHLDA3 deficiency is an important factor in pNET development, but it is not an absolute one. Inactivation of MEN1, another well-known tumor suppressor of pNETs, together with of PHLDA3 is necessary for the development of pNET [15]. In other words, deficiency of PHLDA3 alone induces hyperplasia and increases the insulin-releasing function of islets without inducing pNET. These characteristics potentially make PHDLA3-knockout islets desirable candidates for transplantation.

We have been able to clarify three important findings that support the usefulness and rationality of using PHLDA3-deficient islets for transplantation. First, the viability of PHLDA3-deficient islets was maintained at a higher level after the isolation process. The islet isolation process cannot harvest 100% of the islets from a donor pancreas, and the reagents used during the isolation process and ischemia lead to oxidative stress, which further limits recovery [24]. Our data shows that PHLDA3 deficiency renders islets more resistant to these stresses and prevents damage during the islet isolation process.

Second, we observed that many PHLDA3-deficient islets overcame the stresses encountered in the early stages of transplantation and were successfully engrafted, leading to a dramatic improvement in the metabolic condition of the islets in their new host. Base on earlier work, we had anticipated that PHLDA3-deficient islets would exhibit hyperplasia, which might explain the improved transplantation efficiency; however in the present study we could not detect any difference in the sizes of islets between the KO and WT groups at 56 days after transplantation. This may be due to the early age of the mice from which the islets were harvested: hyperplasia has not been observed for at least 4–5 months after the birth of PHLDA3-deficient mice, but the sizes of islets tends to increase at 10 months after birth. It is known that approximately 60% of transplanted islets fail to engraft in animal models due to the various stresses encountered in the early stages of transplantation [25–27]. Our data showed a marked decrease in apoptosis in the PHLDA3-deficient islets at 12 hs after transplantation, and we found that more islets in the KO group were successfully engrafted by 56 days after transplantation. Thus, we conclude that PHLDA3 deficiency promotes the resistance of islets against stresses at the early stage of transplantation and improves engraftment. In other words, transplantation of PHLDA3-deficient islets improves the metabolic function of diabetic animals by preventing cellular damage at the early stages of transplantation, and by hyperplasia over the longer course (probably more than a year) after transplantation.

**Fig 6:**
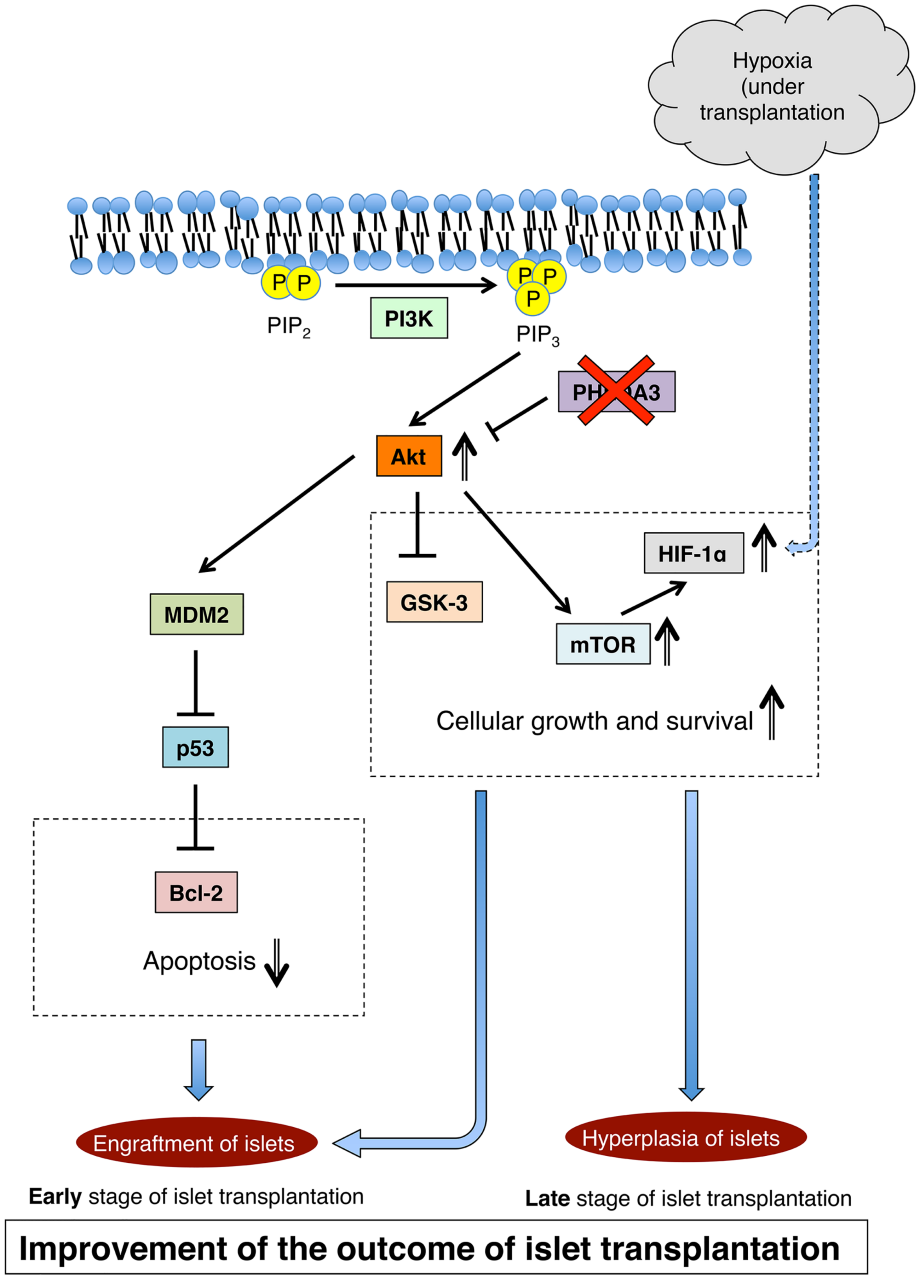
Proposed mechanism underlying the effects of PHLDA3 deficiency on islet transplantation. Modulation of the PI3K / Akt signaling pathway is proposed to be responsible for improved cell growth and reduced apoptosis seen with transplanted PHLDA3-deficient islets under hypoxic conditions.

Third, we observed enhanced activation of Akt in PHLDA3-deficient islets in response to hypoxia. This activation induces the expression of various proteins which are correlated with cellular growth and survival, and with the inhibition of apoptosis. HIF-1α is well known to be strongly expressed under hypoxic conditions. This transcription factor component promotes cellular growth under the regulation of mTOR, which is a downstream factor in the PI3K / Akt signaling pathway [28]. Akt also promotes phosphorylation of GSK3-β, which functions in cytoprotection, promotes an increase in β cell mass, and improves β cell function [29, 30]. Activation of the PI3K / Akt signaling pathway can also suppress apoptosis, as this pathway activates MDM2, which inhibits Bcl-2 via suppression of p53 [31]. Moreover, it is known that this pathway directly suppresses pro-apoptotic Bcl-2 family proteins including Bax, Bim and Bad and prevents apoptosis [32]. Modulation of these pathways presumably underlies the enhanced viability of PHLDA3-deficient islets and prevention of early graft loss (Fig 6).

This study provides mechanistic support for the idea that PHLDA3-deficient islets are an improved source for islet transplantation. These findings support the possibility that transplantation of PHLDA3 knock-down human islets may have therapeutic benefit in the clinic. Further studies using human islets will be necessary to assess this possibility, but may encounter difficulties due to limited donor supplies, especially in Japan. Therefore, we are exploring other novel strategies for utilizing PHLDA3-deficient islets to improve the outcome of islet transplantation. One of the efforts is the development of PHLDA3-knockout large animals. If successful, this effort could help overcome the problem of limited donor supplies and potentially provide a significant improvement in the outcome of islet transplantation. The donor model being developed is a non-human primate, thus this strategy would involve xenotransplantation. However, we are exploring the possibility of combining this with an encapsulation technology that can protect xenogeneic islets from immunity [33, 34], and potentially allow this xenotransplantation model to be used in a future clinical setting. Moreover, we are exploring the possibility of applying PHLDA3 deficiency to stem cell-derived β cells [35–37]. It is known that these cells have a metabolic function, but at present this function is not equal to that of pancreatic islets. We anticipate that PHLDA3 deficiency can also improve the metabolic function of these stem cell-derived β cells, thus allowing them to be utilized as useful donor sources in future.

## Conclusion

Our results show that genetic ablation of PHLDA3 improves islet transplantation due to the prevention of cellular damage at an early stage of transplantation.

## Supporting information

S1 Table Western blot antibodies used in this study. (DOCX)

## References

[1] Fiorina P, Shapiro AM, Ricordi C, Secchi A. The clinical impact of islet transplantation. Am J Transplant. 2008;8(10):1990–7. doi: 10.1111/j.1600-6143.2008.02353.x . 18828765 10.1111/j.1600-6143.2008.02353.x

[2] Fiorina P, Folli F, Bertuzzi F, Maffi P, Finzi G, Venturini M, et al Long-term beneficial effect of islet transplantation on diabetic macro-/microangiopathy in type 1 diabetic kidney-transplanted patients. Diabetes Care. 2003;26(4):1129–36. . 12663585 10.2337/diacare.26.4.1129

[3] Fiorina P, Folli F, Zerbini G, Maffi P, Gremizzi C, Di Carlo V, et al Islet transplantation is associated with improvement of renal function among uremic patients with type I diabetes mellitus and kidney transplants. J Am Soc Nephrol. 2003;14(8):2150–8. . 12874470 10.1097/01.asn.0000077339.20759.a3

[4] Bassi R, Fiorina P. Impact of islet transplantation on diabetes complications and quality of life. Curr Diab Rep. 2011;11(5):355–63. doi: 10.1007/s11892-011-0211-1 . 21748256 10.1007/s11892-011-0211-1

[5] Sakata N, Gu Y, Qi M, Yamamoto C, Hiura A, Sumi S, et al Effect of rat-to-mouse bioartificial pancreas xenotransplantation on diabetic renal damage and survival. Pancreas. 2006;32(3):249–57. doi: 10.1097/01.mpa.0000203959.31877.8c . 16628079 10.1097/01.mpa.0000203959.31877.8c

[6] Fiorina P, Venturini M, Folli F, Losio C, Maffi P, Placidi C, et al Natural history of kidney graft survival, hypertrophy, and vascular function in end-stage renal disease type 1 diabetic kidney-transplanted patients: beneficial impact of pancreas and successful islet cotransplantation. Diabetes Care. 2005;28(6):1303–10. . 15920043 10.2337/diacare.28.6.1303

[7] Shapiro AM, Lakey JR, Ryan EA, Korbutt GS, Toth E, Warnock GL, et al Islet transplantation in seven patients with type 1 diabetes mellitus using a glucocorticoid-free immunosuppressive regimen. N Engl J Med. 2000;343(4):230–8. doi: 10.1056/NEJM200007273430401 . 10911004 10.1056/NEJM200007273430401

[8] Ryan EA, Paty BW, Senior PA, Bigam D, Alfadhli E, Kneteman NM, et al Five-year follow-up after clinical islet transplantation. Diabetes. 2005;54(7):2060–9. . 15983207 10.2337/diabetes.54.7.2060

[9] Mattsson G, Carlsson PO, Olausson K, Jansson L. Histological markers for endothelial cells in endogenous and transplanted rodent pancreatic islets. Pancreatology. 2002;2(2):155–62. doi: 10.1159/000055906 . 12123096 10.1159/000055906

[10] Johansson H, Goto M, Dufrane D, Siegbahn A, Elgue G, Gianello P, et al Low molecular weight dextran sulfate: a strong candidate drug to block IBMIR in clinical islet transplantation. Am J Transplant. 2006;6(2):305–12. doi: 10.1111/j.1600-6143.2005.01186.x . 16426314 10.1111/j.1600-6143.2005.01186.x

[11] Kanak MA, Takita M, Kunnathodi F, Lawrence MC, Levy MF, Naziruddin B. Inflammatory response in islet transplantation. Int J Endocrinol. 2014;2014:451035 doi: 10.1155/2014/451035 24883060 10.1155/2014/451035 PMC4021753

[12] Hata T, Sakata N, Yoshimatsu G, Tsuchiya H, Fukase M, Ishida M, et al Cholestatic Liver Injury After Biliary Reconstruction Impairs Transplanted Islet Viability and Function. Am J Transplant. 2015 doi: 10.1111/ajt.13266 . 25908212 10.1111/ajt.13266

[13] Walters SN, Luzuriaga J, Chan JY, Grey ST, Laybutt DR. Influence of chronic hyperglycemia on the loss of the unfolded protein response in transplanted islets. J Mol Endocrinol. 2013;51(2):225–32. doi: 10.1530/JME-13-0016 . 23833251 10.1530/JME-13-0016

[14] Kawase T, Ohki R, Shibata T, Tsutsumi S, Kamimura N, Inazawa J, et al PH domain-only protein PHLDA3 is a p53-regulated repressor of Akt. Cell. 2009;136(3):535–50. doi: 10.1016/j.cell.2008.12.002 . 19203586 10.1016/j.cell.2008.12.002

[15] Ohki R, Saito K, Chen Y, Kawase T, Hiraoka N, Saigawa R, et al PHLDA3 is a novel tumor suppressor of pancreatic neuroendocrine tumors. Proc Natl Acad Sci U S A. 2014;111(23):E2404–13. doi: 10.1073/pnas.1319962111 . 24912192 10.1073/pnas.1319962111 PMC4060701

[16] Frank D, Fortino W, Clark L, Musalo R, Wang W, Saxena A, et al Placental overgrowth in mice lacking the imprinted gene Ipl. Proc Natl Acad Sci U S A. 2002;99(11):7490–5. doi: 10.1073/pnas.122039999 . 12032310 10.1073/pnas.122039999 PMC124258

[17] Gotoh M, Maki T, Kiyoizumi T, Satomi S, Monaco AP. An improved method for isolation of mouse pancreatic islets. Transplantation. 1985;40(4):437–8. . 2996187 10.1097/00007890-198510000-00018

[18] Kaida A, Miura M. Differential dependence on oxygen tension during the maturation process between monomeric Kusabira Orange 2 and monomeric Azami Green expressed in HeLa cells. Biochem Biophys Res Commun. 2012;421(4):855–9. doi: 10.1016/j.bbrc.2012.04.102 . 22554525 10.1016/j.bbrc.2012.04.102

[19] Xin Y, Jiang X, Wang Y, Su X, Sun M, Zhang L, et al Insulin-Producing Cells Differentiated from Human Bone Marrow Mesenchymal Stem Cells In Vitro Ameliorate Streptozotocin-Induced Diabetic Hyperglycemia. PLoS One. 2016;11(1):e0145838 doi: 10.1371/journal.pone.0145838 . 26756576 10.1371/journal.pone.0145838 PMC4710504

[20] Porciuncula A, Kumar A, Rodriguez S, Atari M, Arana M, Martin F, et al Pancreatic differentiation of Pdx1-GFP reporter mouse induced pluripotent stem cells. Differentiation. 2016 doi: 10.1016/j.diff.2016.04.005 . 27181524 10.1016/j.diff.2016.04.005

[21] Xing BH, Yang FZ, Wu XH. Naringenin enhances the efficacy of human embryonic stem cell-derived pancreatic endoderm in treating gestational diabetes mellitus mice. J Pharmacol Sci. 2016;131(2):93–100. doi: 10.1016/j.jphs.2016.04.014 . 27156928 10.1016/j.jphs.2016.04.014

[22] Morozov VA, Wynyard S, Matsumoto S, Abalovich A, Denner J, Elliott R. No PERV transmission during a clinical trial of pig islet cell transplantation. Virus Res. 2016;227:34–40. doi: 10.1016/j.virusres.2016.08.012 . 27677465 10.1016/j.virusres.2016.08.012

[23] Elghazi L, Bernal-Mizrachi E. Akt and PTEN: beta-cell mass and pancreas plasticity. Trends Endocrinol Metab. 2009;20(5):243–51. doi: 10.1016/j.tem.2009.03.002 . 19541499 10.1016/j.tem.2009.03.002 PMC4456182

[24] Stiegler P, Schaffellner S, Hackl F, Iberer F, Aigner R, Christine B, et al Isoprostanes as markers of oxidative stress-induced cell damage in porcine islet cell isolation. Transplant Proc. 2010;42(5):1618–20. doi: 10.1016/j.transproceed.2009.11.047 . 20620486 10.1016/j.transproceed.2009.11.047

[25] Citro A, Cantarelli E, Piemonti L. Anti-inflammatory strategies to enhance islet engraftment and survival. Curr Diab Rep. 2013;13(5):733–44. doi: 10.1007/s11892-013-0401-0 . 23912763 10.1007/s11892-013-0401-0

[26] Sakata N, Hayes P, Tan A, Chan NK, Mace J, Peverini R, et al MRI assessment of ischemic liver after intraportal islet transplantation. Transplantation. 2009;87(6):825–30. doi: 10.1097/TP.0b013e318199c7d2 . 19300184 10.1097/TP.0b013e318199c7d2 PMC2779521

[27] Sakata N, Obenaus A, Chan N, Mace J, Chinnock R, Hathout E. Factors affecting islet graft embolization in the liver of diabetic mice. Islets. 2009;1(1):26–33. doi: 10.4161/isl.1.1.8563 . 21084846 10.4161/isl.1.1.8563

[28] Van de Velde S, Hogan MF, Montminy M. mTOR links incretin signaling to HIF induction in pancreatic beta cells. Proc Natl Acad Sci U S A. 2011;108(41):16876–82. doi: 10.1073/pnas.1114228108 . 21949366 10.1073/pnas.1114228108 PMC3193251

[29] Fornoni A, Pileggi A, Molano RD, Sanabria NY, Tejada T, Gonzalez-Quintana J, et al Inhibition of c-jun N terminal kinase (JNK) improves functional beta cell mass in human islets and leads to AKT and glycogen synthase kinase-3 (GSK-3) phosphorylation. Diabetologia. 2008;51(2):298–308. doi: 10.1007/s00125-007-0889-4 . 18066521 10.1007/s00125-007-0889-4

[30] Liu Y, Tanabe K, Baronnier D, Patel S, Woodgett J, Cras-Meneur C, et al Conditional ablation of Gsk-3beta in islet beta cells results in expanded mass and resistance to fat feeding-induced diabetes in mice. Diabetologia. 2010;53(12):2600–10. doi: 10.1007/s00125-010-1882-x . 20821187 10.1007/s00125-010-1882-x PMC2991091

[31] Chang H, Li C, Huo K, Wang Q, Lu L, Zhang Q, et al Luteolin Prevents H2O2-Induced Apoptosis in H9C2 Cells through Modulating Akt-P53/Mdm2 Signaling Pathway. Biomed Res Int. 2016;2016:5125836 doi: 10.1155/2016/5125836 . 27525270 10.1155/2016/5125836 PMC4976196

[32] Wang S, Chong ZZ, Shang YC, Maiese K. Wnt1 inducible signaling pathway protein 1 (WISP1) blocks neurodegeneration through phosphoinositide 3 kinase/Akt1 and apoptotic mitochondrial signaling involving Bad, Bax, Bim, and Bcl-xL. Curr Neurovasc Res. 2012;9(1):20–31. . 22272766 10.2174/156720212799297137 PMC3274638

[33] Yang KC, Qi Z, Wu CC, Shirouza Y, Lin FH, Yanai G, et al The cytoprotection of chitosan based hydrogels in xenogeneic islet transplantation: An in vivo study in streptozotocin-induced diabetic mouse. Biochem Biophys Res Commun. 2010;393(4):818–23. doi: 10.1016/j.bbrc.2010.02.089 . 20171166 10.1016/j.bbrc.2010.02.089

[34] Yang KC, Wu CC, Lin FH, Qi Z, Kuo TF, Cheng YH, et al Chitosan/gelatin hydrogel as immunoisolative matrix for injectable bioartificial pancreas. Xenotransplantation. 2008;15(6):407–16. doi: 10.1111/j.1399-3089.2008.00503.x . 19152669 10.1111/j.1399-3089.2008.00503.x

[35] Massumi M, Pourasgari F, Nalla A, Batchuluun B, Nagy K, Neely E, et al An Abbreviated Protocol for In Vitro Generation of Functional Human Embryonic Stem Cell-Derived Beta-Like Cells. PLoS One. 2016;11(10):e0164457 doi: 10.1371/journal.pone.0164457 . 27755557 10.1371/journal.pone.0164457 PMC5068782

[36] Miladpour B, Rasti M, Owji AA, Mostafavipour Z, Khoshdel Z, Noorafshan A, et al Quercetin potentiates transdifferentiation of bone marrow mesenchymal stem cells into the beta cells in vitro. J Endocrinol Invest. 2016 doi: 10.1007/s40618-016-0592-8 . 28000178 10.1007/s40618-016-0592-8

[37] Konagaya S, Iwata H. Reproducible preparation of spheroids of pancreatic hormone positive cells from human iPS cells: An in vitro study. Biochim Biophys Acta. 2016;1860(9):2008–16. doi: 10.1016/j.bbagen.2016.05.012 . 27180174 10.1016/j.bbagen.2016.05.012

