# Supplementary material for: Pleckstrin homology-like domain family A, member 3 (PHLDA3) deficiency improves islets engraftment through the suppression of hypoxic damage
Source: PLoS One. 2017 Nov 9;12(11):e0187927. doi: 10.1371/journal.pone.0187927 (PMC5679611; doi:10.1371/journal.pone.0187927)
Supplement: S1 Table — (DOCX) [file pone.0187927.s001.docx]

**S1 Table. Western blot antibodies used in this study**

| **Primary antibody** | **Dilution** | **Buffer** | **Reference** | **Provider** | **Secondary antibody** |
| --- | --- | --- | --- | --- | --- |
| **pAkt (Ser473)** | 1:2,000 | Signal Enhancer Hikari (solution A) | clone D9E  #4060 | Cell Signaling Technology | Anti-Rabbit -HRP |
| **pAkt (Thr308)** | 1:1,000 | Signal Enhancer Hikari (solution A) | #9275 | Cell Signaling Technology | Anti-Rabbit -HRP |
| **Akt** | 1:3,000 | PBST with 3%BSA | #4691 | Cell Signaling Technology | Anti-Rabbit -HRP |
| **pMDM2 (Ser166)** | 1:1,000 | Signal Enhancer Hikari (solution A) | #3521 | Cell Signaling Technology | Anti-Rabbit -HRP |
| **MDM2** | 1:1000 | Signal Enhancer Hikari (solution A) | SMP-14 | Santa Cruz | Anti-Mouse -HRP |
| **pGSK-β** | 1:1,000 | Signal Enhancer Hikari (solution A) | clone D75D3  #5676 | Cell Signaling Technology | Anti-Rabbit -HRP |
| **GSK3-β** | 1:1,000 | Signal Enhancer Hikari (solution A) | clone 3D10  #9832S | Cell Signaling Technology | Anti-Mouse  -HRP |
| **HIF-1α** | 1:1,000 | Signal Enhancer Hikari (solution A) | Ab179483 | Abcam | Anti-Rabbit –HRP |
| **pp70 S6 kinase**  **(T389)** | 1:1,000 | Signal Enhancer Hikari (solution A) | #9305 | Cell Signaling Technology | Anti-Rabbit -HRP |
| **p70 S6 kinase** | 1:1,000 | Signal Enhancer Hikari (solution A) | clone 49D7  #2708S | Cell Signaling Technology | Anti-Rabbit –HRP |
| **p4E-BP1**  **(T37/46)** | 1:1,000 | Signal Enhancer Hikari (solution A) | clone 236B4  #2855S | Cell Signaling Technology | Anti-Rabbit –HRP |
| **4E-BP1** | 1:1,000 | Signal Enhancer Hikari (solution A) | #9452 | Cell Signaling Technology | Anti-Rabbit –HRP |
| **β-actin** | 1:5,000 | PBST with 3%BSA | clone AC-74  A2228 | SIGMA | Anti-Mouse –HRP |
| **β-actin** | 1:1,000 | PBST with 3%BSA | clone C4 | EMD Millipore | Anti-Mouse –HRP |
| **β-actin** | 1:3,000 | PBST with 3%BSA | clone C4 | Santa Cruz | Anti-Mouse –HRP |
